# Integrated Video and Acoustic Emission Data Fusion for Intelligent Decision Making in Material Surface Inspection System

**DOI:** 10.3390/s22218554

**Published:** 2022-11-06

**Authors:** Andrey V. Chernov, Ilias K. Savvas, Alexander A. Alexandrov, Oleg O. Kartashov, Dmitry S. Polyanichenko, Maria A. Butakova, Alexander V. Soldatov

**Affiliations:** 1 The Smart Materials Research Institute, Southern Federal University, 178/24 Sladkova, 344090 Rostov-on-Don, Russia; 2 School of Technology, University of Thessaly, Larissa-Trikala Ring-Road, 415000 Larissa, Greece

**Keywords:** data fusion, intelligent system, decision making, computer vision, acoustic emission control, surface inspection

## Abstract

In the field of intelligent surface inspection systems, particular attention is paid to decision making problems, based on data from different sensors. The combination of such data helps to make an intelligent decision. In this research, an approach to intelligent decision making based on a data integration strategy to raise awareness of a controlled object is used. In the following article, this approach is considered in the context of reasonable decisions when detecting defects on the surface of welds that arise after the metal pipe welding processes. The main data types were RGB, RGB-D images, and acoustic emission signals. The fusion of such multimodality data, which mimics the eyes and ears of an experienced person through computer vision and digital signal processing, provides more concrete and meaningful information for intelligent decision making. The main results of this study include an overview of the architecture of the system with a detailed description of its parts, methods for acquiring data from various sensors, pseudocodes for data processing algorithms, and an approach to data fusion meant to improve the efficiency of decision making in detecting defects on the surface of various materials.

## 1. Introduction

Human vision is a unique ability that allows us to see and recognize a variety of objects in dynamic environments. Vision provides us with essential information that helps us make intelligent and rational decisions in many areas (such as quality inspection). An experienced specialist can easily visually distinguish whether a surface of material has exemplary quality or not. The surfaces of metal pipelines are no exception in this case. It is certain that a lot of a viewer’s attention will be focused on the surface of welding seams [1] because most of the defects [2] will be concentrated in those regions. Long metal pipelines are almost always manufactured using various seam welding technologies [3]. Therefore, the surfaces of pipelines must be inspected after being joined. In some cases, weld integrity defects can be easily detected on the surface. Experienced experts can distinguish porosity, crater cracks, incomplete fusion, underfill, undercut, and incomplete joint penetration via visual inspection (and, in certain cases, under slag). Different types of weld defects cause a limited pipeline lifetime, but their levels of influence are not equal in the general sense. Due to this fact, visually detected surface defects should usually be classified according to the type of welding and pipeline fabrication method used. This field has a wide diversity. For example, the recommended defect classifications for fusion welding quality and imperfections in metallic material joints are given in ISO 6520-1:2007 standard (Welding and allied processes—Classification of geometric imperfections in metallic materials—Part 1: Fusion welding) and ISO 5817:2014 standard (Welding—Fusion-welded joints in steel, nickel, titanium, and their alloys (beam welding excluded)—Quality levels for imperfections).

It is obvious that the early detection of surface defects helps to increase the pipeline production quality, avoid failures in mechanical pipeline components, ensure a higher quality production, and reduce cost and time expense for maintenance and repair. Visual inspection of the welding joints on the metal pipeline surface alone cannot replace other advanced nondestructive testing (NDT) techniques [4]; however, it remains an important first stage for a deeper examination of the quality of the pipeline. NDT techniques [5] include a range of integrity analysis approaches that assess the properties of the material from which the pipeline is made. The most used techniques are: ultrasound testing [6,7,8], including phased array ultrasonic testing [9,10]; radiography testing [11,12]; magnetic particle and other flux leakage-based inspections [13]; approaches based on acoustic emission [14,15,16]; hybrid testing methods [17]; and several other methods.

The surface quality of pipelines containing welded seams is being inspected using artificial intelligence (AI) and machine learning (ML) technologies more and more often [18]. We should note that material surface inspection and surface weld quality assessment using AI and ML is a complex, data-intensive process influenced by many factors [19]. However, recent studies show that the AI and ML approaches to weld quality analysis are capable of detecting, locating, and classifying different anomalies on the pipeline surface, especially for steel materials. A review of ML approaches in pipeline condition assessment, including computer vision-based methods for the detection, localization, and quantification of surface anomalies, is presented in [20]. This review shows that researchers intensively use different categories of ML techniques for assessing the condition of pipelines and their surfaces. An approach to weld defect classification based on radiographic testing data and deep learning has been presented in [21]. The authors proposed an end-to-end semantic segmentation-based solution for the heat-affected weld zone and then applied Gaussian filtering based on a deep belief network. Their results showed high classification precision (ranging from 79.9% to 99.8%) for several categories of defects (crack, porosity, inclusion of slag, and background). Another approach to detecting defects by classifying them into 11 categories (elongated slag inclusion, hollow bead, isolated slag inclusions, gas porosity, inadequate penetration, external undercut, porosity, scattered porosity, inadequate penetration due to high-low, internal undercut, and internal concavity) using deep convolutional neural networks (DCNNs) and two databases with patches of radiographic testing data labeled defective and nondefective was proposed in [22]. In the experimental study, the authors used several DCNN architectures (AlexNet, ResNet, and VGG-based) and reported that the overall accuracy reached 96% of overall classes.

Despite the seeming prevalence of products made via welding parts, the problem of collecting data sets containing instances of weld images with labeled and classified defects is still difficult. The most well-known public database with X-ray images partly consisting of welds is GDXray [23], which contains up to 20,000 images in total for solving NDT tasks. The welds portion of the GDXray database is not that large and includes less than 80 images. It is obvious that the limited quantity of images complicates the applicability of newer DCNN architectures for vision detection and classification of weld defects while lowering the detection rate. A less famous NDT dataset, which consists of 13,766 X-ray images with valid annotation, is the WDXI dataset [24]. We should note that one of the approaches to making DCNN detection performance higher and more reliable is extending the small number of weld samples via data augmentation techniques. Such a technique, using GAN (Generative Adversarial Network), has been proposed in [25].

Due to the limited datasets with weld defects samples, two problems are at the center of the research interest, namely (1) feature extraction and (2) fine-tuning strategies to improve and obtain better generalization performances from the applied DCNNs. The main categories of features to be extracted from weld defect images include the geometric, intensity, and contrast properties of the welds. The authors in [26] extracted 11 features within the above-mentioned types (length, aspect ratio, sharpness, roughness of defect edge, roughness of defect region, skewness, kurtosis, histogram contrast, roughness contrast, skewness contrast, and kurtosis contrast). Then, they applied a unified deep neural network with multilevel features, pretraining, and fine-tuning training strategies in the workflow of defect classification. The dataset in [26] was relatively small at 220 samples (with 176 samples in the training set and 44 samples in the test set), but for the whole model, the average training accuracy was 97.95% and the average testing accuracy was 91.36%. CNN feature extraction and a deep learning-based classification of weld surface defects were previously discussed in [27].

The authors of [28] also noted that the detection of weld defects in small datasets is a difficult and complex mission. They chose a pretrained AlexNet architecture, the blocks of which contain a convolutional layer, a cluster layer, and rectified linear units (ReLU), followed by a fully connected (FC) layer and Softmax classification. The training of such a DNN architecture took 1 min on GPU GeForce RTX 2080, with 80 iterations run within 10 training epochs. After evaluation of the AlexNet results, they performed fine-tuning of the architecture using a comparison of transfer learning-based pretrained models beyond AlexNet (VGG-16, VGG-19, GoogLeNet, ResNet50, and ResNet101). The results showed accuracy, precision, sensitivity, and specificity up to 100%, which translates to no weld defect recognition errors (0% error).

In our study, we not only used X-ray or standard RGB camera images but also augmented the data with acoustic emission (AE) sensor technology. Let us briefly overview the applications of AE for the classification of weld defects. AE defects recognition is a type of inner NDT technique. The AE NDT technique is an important practice for assessing the material integrity of welded structures. The signals of AE control are capable of recognizing, at minimum, cracks, slack inclusion, and porosity in welded joints [29]. The AE signal has many informative features, but its processing is not very trivial. Firstly, it is a wide range signal (ranging from 50–800 kHz) and therefore special acoustic spectrum transforms should be used to analyze it; for example, using Fourier transforms for its screening [30]. Secondly, the dissimilarity in the steel and weld materials can cause noise disturbance in the typical AE signal waveforms [31]. Consequently, the standard analysis (such as FFT) in the frequency domain may be noninformative. Thus, the time-frequency domain analysis and the most practical wavelet techniques for the detection of nonstationary components that are present in the AE signal can be successfully applied [32]. In a similar manner to the visual inspection data processing, AE data processing may be performed based on machine learning (ML) and DCNNs. In [33], the methodology for weld quality monitoring with acoustic signature and classification results using the ML random forest algorithm was proposed. A deep learning approach to processing AE signals was proposed in [34], wherein the authors applied the continuous wavelet transform scalograms based on CNN architecture.

In summarizing this introduction, we should note that the approaches listed above are separately aimed at outer and inner defect detection. Indeed, visual inspection makes it possible to detect surfaces and their geometry form defects only, but nondestructive approaches provide information about the inner condition of the tested material while saying nothing about the geometric form of the investigated surface. Nevertheless, visual information and information provided by nondestructive testing can be integrated to ensure improvement in decision making procedures. In other words, if the visual inspection of the welded joints (RGB-Depth images) is augmented with AE NDT, then that inspection accelerates automated and intelligent pipeline surface inspection problem-solving. 

**Figure 1:**
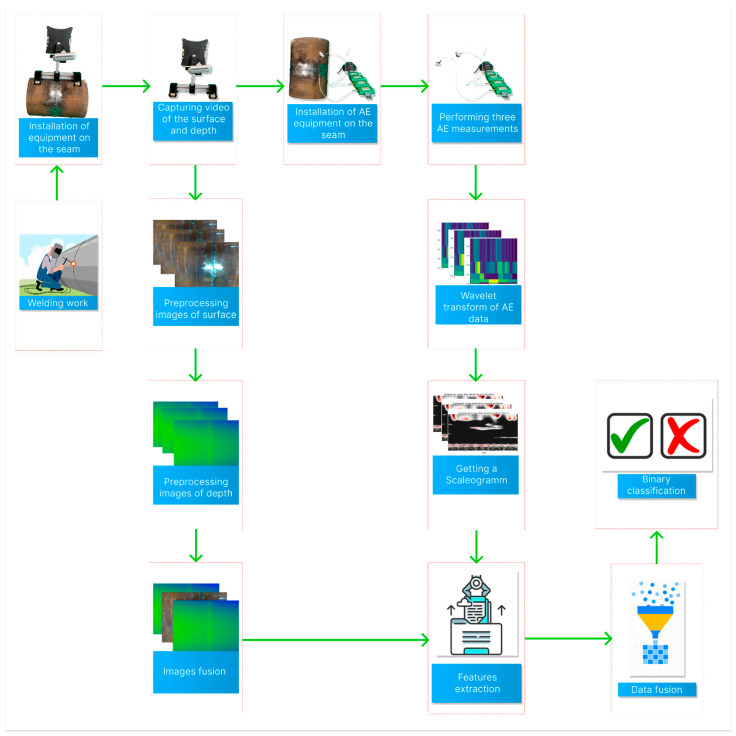
The block diagram of the proposed study.

The contribution of our research is as follows: firstly, we propose a novel approach to correctly combining the data obtained from visual inspection using an RGB-Depth camera and an AE setup using sensors. Then, we present and describe this approach in the form of a surface inspection system with intelligent decision making capabilities. The general block diagram of the proposed study is introduced in Figure 1.

The details of the experimental setups, dataset samples, DCNN architectures, and source codes for the proposed approach are found in the following repositories: (1) the main repository, which contains fusion techniques and is located at https://github.com/cybervllc/weldfs (accessed on 30 September 2022); (2) the details of the RGB-Depth data acquired based on web interface from an Intel RealSense D435i camera, which are located at https://github.com/cybervllc/weldrs (accessed on 30 September 2022); and (3) the details of AE processing and the samples of AE data, which are located at https://github.com/cybervllc/acoustic_emission (accessed on 30 September 2022).

## 2. Materials and Methods

**Figure 2:**
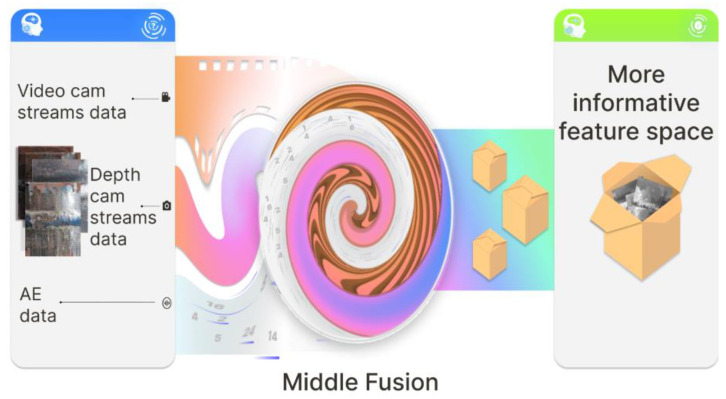
The used raw data streams and the principles of their fusion.

This section describes essential aspects regarding research approaches that have been used to obtain results. Here, we will briefly explain the foundational idea of our research. As previously discussed in the introduction, the vast majority of surface inspection systems are constructed using some type of sensor depending on the accessibility of the surface under inspection and the material that was made with its internal properties. In choosing a particular sensor, the researcher should be prepared for the restrictions that follow with the selected sensor type: for instance, if the video sensor is chosen, then the visual image processing technique later will be applied later, and if the AE sensor is chosen, then one of the AE signal processing approaches will be applied. It is worth noting that the image and AE processing approaches are not interchangeable, nor do they demonstrate a high degree of similarity in the general sense. However, in the real world and in nature, many “sensors” of human beings or animals are rather different and give an informative environmental picture that cannot be obtained using one sensor type only. The information fusion process thus occurs in the brain activity. In a similar manner to natural information fusion, our study is based on obtaining and consolidating heterogeneous data streams, as shown in Figure 2. In the future, this will provide a more informative feature space.

According to Figure 2, the developed inspection system has three channels with different sensors, including (1) RGB image sensors, (2) depth data sensors, and (3) AE signal sensors. After obtaining the data streams, they are preprocessed and the middle fusion principle is then applied. The technical details of the sensors and the full design of the developed system will be revealed in the results section. The proposed inspection system provides intelligent recommendations about the presence of defects in the steel pipeline containing the welded joints. For clarification of the future discussion, it should be noted that our system realizes two class weld defect classifications because the main contribution of this research is the novelty and originality of the data fusion system architecture. We think the proposed system architecture can be modified for true multiclass weld defect classification rather easily. 

### 2.1. Data Acquisition

First, we describe a mechanical part of our inspection system, which carries electronics and power elements. The main elements of the structure on which the system is installed in our case are steel metal pipes of different diameters connected with welded joints between them, obtained via electric arc welding. Among the fasteners and restraints of other structures on metal pipes, we can note the predominance of devices based on flexible rails. For example, a TecScan manual time of flight diffraction with a pipe scanner configuration can exist. Another example is the Rotix corrosion chain scanner for NDT pipe inspection. This type of design usually consists of fixed segments that allow the length of the rail to be adjusted to the diameter of the steel pipe by using a set of segments and connecting them to each other. The flexible rail guide itself can be secured to the pipe as a clamp or by means of permanent magnets. The advantages of this approach include a reliable fixation of the structure, as well as durability and accuracy of movement of the device along the rail. The disadvantages of a design using a flexible rail are the complexity of installation (as it requires time-consuming assembly and disassembly of the guideway), the difficulty of purchasing flexible rail components, and its high cost.

**Figure 3:**
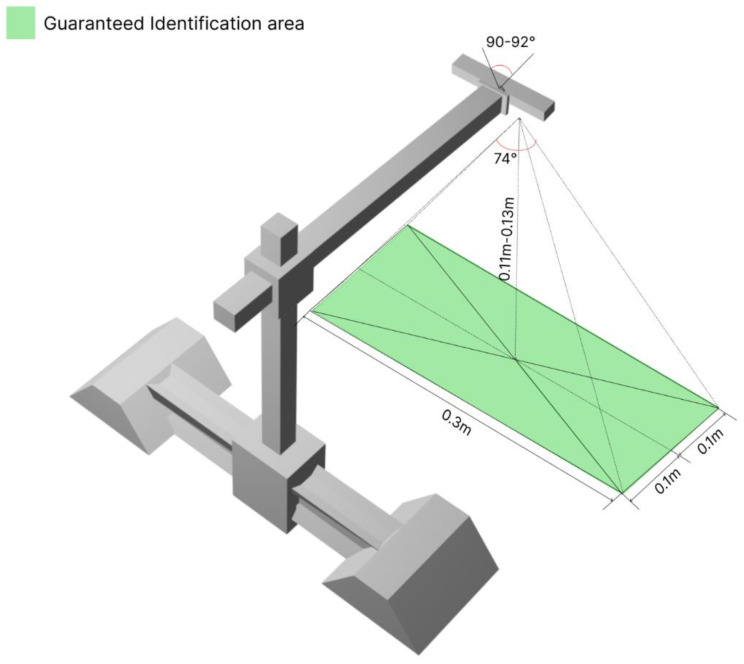
Parameters of a guaranteed identification area above the steel pipe surface.

For visual inspection using an RGB camera and with various depth estimation approaches, the guaranteed identification area at the steel pipe surface inspection should be identified using the geometric parameters shown in Figure 3.

The system prototype used an Intel RealSense D435i camera (which has a built-in IMU sensor and allows for both indoor and outdoor use) to capture images and video streams. With a built-in active infrared stereo projector, the camera does not need any additional lighting setup and allows the user to estimate the depth of a surface. The minimum working distance of the camera is about 28 cm, which corresponds to the dimensions of the guaranteed identification area shown in Figure 3 when set up using the overall dimensions of the camera itself (90 × 25 × 25 mm). The camera weight is 0.4 kg and has a standard fixing mechanism, which is why a standard hinge mount was used for its installation on the system prototype.

**Figure 4:**
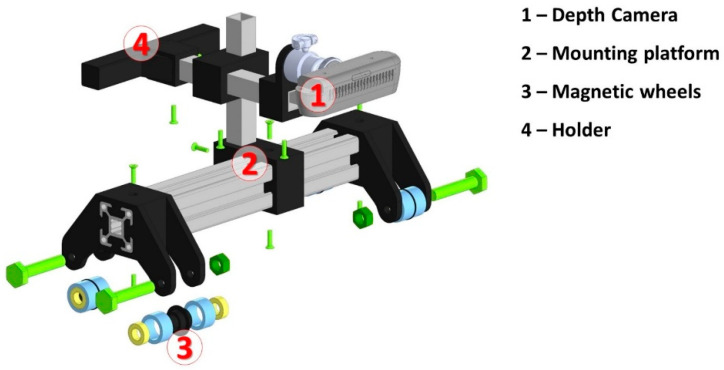
A 3D mechanical prototype model with an installed RGB-Depth camera and magnetic wheels.

As a fixture for adhering the holding device onto the steel pipe, a design based on neodymium magnetic wheels was chosen. This solution allows for fast fixation of the prototype in the pipe, and the use of rubber coating allows for the exclusion of displacement relative to the line of removal. Neodymium ring magnets 20 × 15 × 5 mm in size were used in the magnetic wheels. Two neodymium alloy N38 ring magnets with a protective nickel coating were used for each wheel. The weight that the magnet withstands depends on each factor separately, as the dependence is made up of such factors as: the manner of fixing the magnet, the thickness and roughness of the metal, the area of the magnet, and the presence of a gap between the material and the magnet. The maximum bonding force of the magnet is achieved when it adheres to the metal with a thickness of at least 20 mm. A 3D model of the proposed prototype construction with an Intel RealSense D435i RGB depth camera installed, the construction body, and the magnetic wheels is presented in Figure 4.

Such a system construction allowed us to provide two data channels: (1) RGB and (2) depth data streams captured in real time from the inspected weld seam area aimed to ensure NDT tasks.

The third data channel was an AE signal. AE is a phenomenon associated with the emergence and propagation of sound vibrations (elastic vibrations, sound waves, etc.) in a solid medium for a structural material subjected to deformation due to mechanical failure, as well as other deformations. A unique feature of AE is a steadily distinguishable sound sequence, which has characteristic features for different materials and different varieties of deformations in those materials. It allows for quantitative assessment of a wide range of deformation changes in materials (cracks, fractures, fractures, delamination, etc.). It is worth noting that practically all deformation processes in a material (including electrochemical and chemical transformations, plastic deformations due to temperature, pressure, friction, and wear) are accompanied by the emergence and propagation of AE. Thus, it is possible to monitor the dynamics of material transformation processes, including the movement of cracks, the development of delamination, and the increase of fractures, considering not just the mechanical causes. At the same time, the propagation of the AE signal can be recorded at tens of meters from the site of defect occurrence.

**Figure 5:**
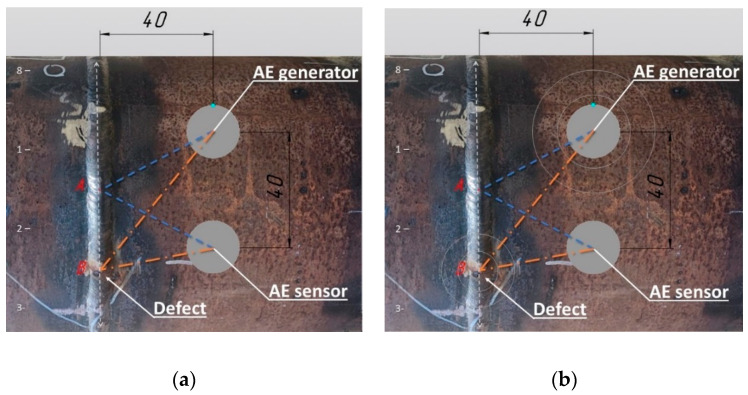
Positioning of the AE sensors on the surface of the pipe near the weld seam: (**a**) geometric parameters; (**b**) AE waves spread from the AE generator to the AE sensor.

Despite AE methods being volume sensitive, the correct determination of the placement of the weld defect directly depends on accurate sensor placement. In our research, these aspects are detailed in Figure 5.

To correctly collect AE signals, we propose the following method: the AE generator and the AE sensor are placed at the same distance from the weld seam and from each other. The distance in Figure 5 is 40 mm. The seam surface is then divided by up to eight imaginary equal lines. Then, we start the AE generator and move some sensors near the seam surface, keeping the selected distance between the sensors and the seam. One of the sensors is used to generate AE signals. The AE signal generator generates waves with a given frequency and power every 2 s. Its AE signal generation parameters are always known in advance. The AE generator produces waves every 2 s, and the sensor collects them at a 300 kHz frequency. For a steel pipe, the wavelength is approximately 19.8 mm and the closest wave path is two periods, which is approximately 40 mm. In Figure 5, this is the shortest path from the AE generator to point A. This AE screening method facilitates the recognition of a signal disruption within a 40 mm distance, which would be a defect probably located at point B. The collected AE signals are saved in the time series arrays. In this study, direct AE sensors were used. The generator produces pulses by creating longitudinal waves in carbon steel. The wavelength was calculated based on the longitudinal wave speed in the steel and the frequency at which the generator was working. We used a pulse generator and an AE receiver with a known distance between them. We started from the steel pipe thickness analyzed, setting the pulse generator frequencies in accordance with the wavelength calculations necessary to exclude standing waves. The signal received from the generator to the sensor was taken directly at the beginning of the measurement, and its characteristics and parameters made up the measured signal constant component; then, the reflected signal from defects inside the weld was added. The defect detection sign is a change in the amplitude of the signal reflected from the defect inside the weld. The further we moved the sensor with the receiver away from the seam, the less the signal from the generator and the reflected signal overlapped. Then, we divided the signal into the generated wave from the generator and the reflected signal from the defect. The collected signal contains data received from the generator; further signal-changing data were received from defects inside the weld. The incident wave on the defect is partially reflected and changes its parameters. The transformed wave from the defect is captured by the AE receiver. The task of the system is to determine the change in signal amplitude due to the reflected signal.

### 2.2. Data Preprocessing

**Figure 6:**
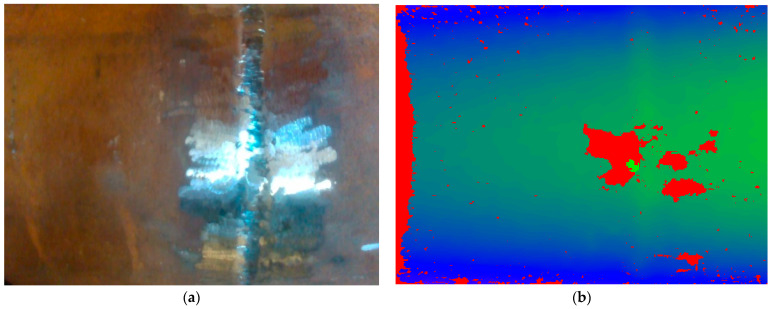
A sample pair of images in the collected dataset: (**a**) an RGB image; (**b**) a corresponding depth image.

As mentioned above, the main RGB-Depth sensor was an Intel RealSense D435i camera that provided RGB and depth real-time streams at 1280 × 768 RGB and 640 × 480 depth resolution. The pipeline of obtaining videoframes from that camera consists of several steps: (1) Obtaining the video and depth profile according to the selected resolution; (2) making a profile for a virtual stream; (3) waiting for an RGB frame and converting it to a numerical 2D array; (4) waiting for the depth frame, imposing the disparity, spatial, and temporal filters, and then converting the filtered depth frame into a 2D numerical array; (5) creating a colored RGB image from the 2D array using the stage 3 function; and (6) applying the color map to the 2D depth arrays and creating an RGB-Depth image. A sample of preprocessed images in our dataset is shown in Figure 6.

AE timeseries were preprocessed with the following method, which is based on the wavelet transform and is required for precise representation of the set of local frequency features in collected AE timeseries. The mathematical apparatus of any wavelet transform method is based on the decomposition of a discrete signal into a basis of special functions called wavelets. A wavelet is then subjected to a number of mathematical constraints related to the symmetric character of the function, the exact localization in the time and frequency representation, the boundedness, and the zero mean. These properties allow us to consider a wavelet as a bandpass filter applied to an AE signal. Symbolically, the integral continuous wavelet transform of the AE timeseries, defined as x(t), can be written as

(1) $$C_{a,b}=\int_{-\infty }^{\infty }x\left(t\right)\psi^{*}\left(\frac{t-b}{a}\right)dt$$

where $C_{a,b}$—wavelet coefficients, $\psi^{*}\left(\frac{t-b}{a}\right)$—specific wavelet function, and a,b—scale and shift parameters.

The set of specific functions $\psi^{*}\left(\frac{t-b}{a}\right)$ with different a,b parameters form the basis for signal transformation, and parameter a practically defines a range of analyzed frequencies. The application of such an approach to preprocessing allows one to perform a frequency–time analysis of an AE signal (timeseries). The initial signal (timeseries) is represented by a set of coefficients on each scale of transformation. The local features of the analyzed timeseries, expressed in the form of changes in amplitude and frequency, find their response in the change of coefficient values at a certain scale and time position. This represents a certain advantage of wavelet analysis over methods based on the Fourier transform method. Based on the analysis of the wavelet transform results, it is possible to not only determine the frequency characteristics of the signal but also establish the moment of the signal frequency change.

We should note that the Equation (1) cannot be applied directly for a discrete time series. In the discrete case, the wavelet transform can be applied through a convolution operation:

(2) $$W\left(j,k\right)=\overset{N-1}{\underset{n=0}{\sum }}x\left[n\right]\psi_{j,k}^{*}\left[n\right]=x⊙\psi_{j,k}^{*}$$

where x[n] and $\psi_{j,k}^{*}$ have discrete forms of timeseries and wavelet function, respectively, and ⊙ is a convolution symbol.

In practice, for a real-time discrete series, Equation (2) has a high computational cost; therefore, an approximated-detailed discrete signal x(t) representation is preferred:

(3) $$x\left(t\right)=A_{m}\left(t\right)+\sum_{i=1}^{m}D_{j}\left(t\right),$$

where $A_{m}\left(t\right)$ represents an approximated averaged part and $D_{j}\left(t\right)$ is a detailed part of a signal considered to be local features at the scale m.

For an AE signal (timeseries), the representation of the wavelet transform according to Equation (3) can be written as:

(4) $$x\left(t\right)=\sum_{j.k}a_{j,k}\varphi_{j,k}\left(t\right)+\sum_{j.k}d_{j,k}\psi_{j,k}\left(t\right)$$

where $a_{j,k},d_{j,k}$ are the approximated and detailed coefficients of j-th level.

**Figure 7:**
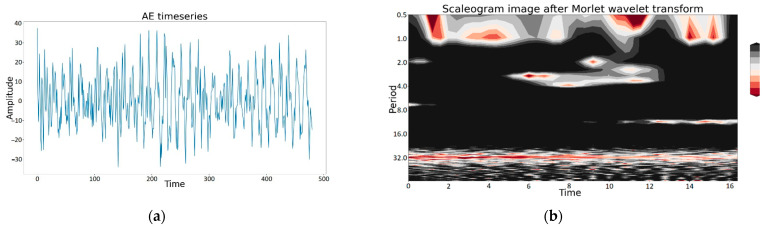
A sample scaleogram image. For input timeseries: (**a**) an input AE timeseries; (**b**) a corresponding scaleogram image.

Due to integrated data fusion, AE timeseries were transformed with the described method, and the resulting output was the dataset containing graphical wavelet representation as a set of scaleograms (in the form of images). A sample scaleogram image from the dataset after Morlet wavelet transform and its corresponding AE timeseries are shown in Figure 7a,b, respectively.

The described data preprocessing generates three input datasets for the data fusion strategy, which is the next step of the proposed integrated video and acoustic emission data fusion approach.

### 2.3. Data Fusion Strategies

Data consolidation, also called data fusion [35] (which belongs to information processes), aims to mimic the natural ability of humans to integrate information from different senses. The consolidation of data from multiple sensors [36] processes heterogeneous data more efficiently to improve accuracy and reliability, reduce uncertainty, and improve information quality. The concept of data fusion has been studied for a long time [37] and offers many different methods aimed at estimating the probabilities of distributed sensor states as well as decision support methods for automated real-time multisensor data fusion and analysis [38]. When acquiring new knowledge about information processes, data fusion is associated with automated methods for extracting meaning from incoming information, selecting algorithms and methods, evaluating information quality, and evaluating information fusion systems. The evaluation of information fusion systems [39] is seen as the biggest challenge; there are still no standard metrics used, so researchers develop their own metrics. We emphasize that the main goal of data consolidation systems always remains the same—to obtain better information about an object, process, or phenomenon, using data from multiple sources.

Data consolidation can be carried out in a variety of ways and can be implemented with different technical solutions. Nevertheless, the focus of any data consolidation process is to produce consolidated data in a single repository, e.g., in the cloud [40]. Data consolidation is based on the data fusion model, which can be roughly divided into high-level information fusion (high-level fusion) and low-level data fusion. Among the high-level data-based fusion models, the best known model is the joint directors of laboratories (JDL) functional model [41]. This model is not suitable for direct implementation in information systems, but theoretically, JDL is very productive, because it enabled a number of advancements such as state transition data fusion (STDF) [42], data fusion information group (DFIG), and others. Depending on the level of abstraction, high-level fusion methods refine the characteristics of information processes, obtain new attributes of information objects and resources, evaluate information situations, and, ultimately, improve solutions.

In our system, there are three image modalities that can be fused using one of the families’ multimodality fusion approaches [43]: early fusion, intermediate fusion, and late fusion. The strategy depends on the stage at which the fusion is performed. In the early fusion strategy, several input streams are combined before the feature extraction procedure. In the intermediate fusion strategy, features are extracted and fused before classification. The late fusion strategy involves fusion of the results after classification, but before a decision making procedure.

### 2.4. Deep Learning Architecture for Multimodal Fusion

The main purpose of selecting a deep architecture to represent the weld defect feature space signs is obtaining extra information about the visual condition of the weld and its geometric and morphological parameters, as well as its internal structure.

We recall that an AE approach was chosen to characterize the internal structure of the pipeline and an intelligent sensor (RGB-Depth camera) was used to obtain a surface depth map. Therefore, we set up a set of morphological descriptions of steel pipeline weld joints. To fully exploit all the features of the data when detecting defective welds using deep learning models, we needed to provide data fusion by enriching feature levels. In our study, we chose the late data fusion approach. We dealt with three different types of images, so we were able to use the same feature extractor model for their subsequent superposition and enrichment of the representation space for reliable intelligent identification of the inspected surface with a small amount of starting data for the classifier model. We used a pretrained ResNet18 as a feature extractor backbone architecture.

This choice was made for several reasons. The ResNet18 has high accuracy while having the experience of its successful application for extracting features from small rigid objects in complex images [44,45]. ResNet-8 model blocks were used directly because, in fact, the 18-level network is a subspace of the 34-level network (ResNet-34) with the same output vector size (512). It has less complexity and has the functionality to bypass two successful convolutional layers, rather than three (as do the more multilevel residual network models). The double depth of the block allows for a more efficient organization of the subsequent fusion of the three image data types, avoiding excessive resource costs while allowing quality feature extraction from all the data types presented. To confirm the effectiveness of the approach proposed in this paper, the ResNet-18 model was also used as a classifier for the individual image types of the control object. We also modified the final classifiers for training the feature embeddings extracted from ResNet-18. In particular, the activation function for convolutional layers was changed from the rectifier linear unit (ReLU) to the Swish-based sigmoid linear unit (SiLU). This choice was made for the sake of increasing the final accuracy with an insignificant increase in computational complexity [46,47]. In the general case, Swish is an extension of the SiLU function, considering the parameter β calculated in the training process. The formula is represented as follows:

(5) $$f\left(x\right)=x*\frac{1}{1+e^{-\beta x}}$$

**Figure 8:**
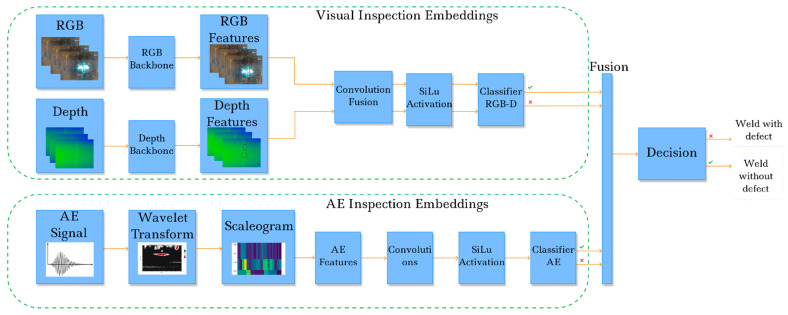
A proposed pipeline for the integrated data fusion with the decision making procedure.

In our case, with representations of weld images, depth images, and acoustic emission data wavelet transform scaleograms, it is important that the subsequent layers of classifiers can propagate negative weights to improve the final accuracy. This possibility is due to the shape of the smooth continuous Swish function in the SiLU. Furthermore, the parameter β allows us to better adapt the activation function to optimize the background of generalization. A general pipeline of the proposed data fusion method used to form an extended feature space and provide training for a reliable and high-quality DCNN for weld defect classification on a small dataset is shown in Figure 8.

The potential of the technology proposed in this study is not limited to use in the nondestructive testing framework of welded joints in steel pipelines. The results obtained can be extrapolated to other critical and key facilities and processes in industry and production. Furthermore, the approach proposed in this article can provide additional opportunities for the early diagnosis of induction motor faults [48] using the intelligent analysis of AE signals and visual control of individual units. In general, the individual modalities data emerging from various sensory devices to enrich the features levels characterizing the controlled object state significantly increase the awareness, reliability, and completeness of decisions made when using deep neural network models.

## 3. Results

**Figure 9:**
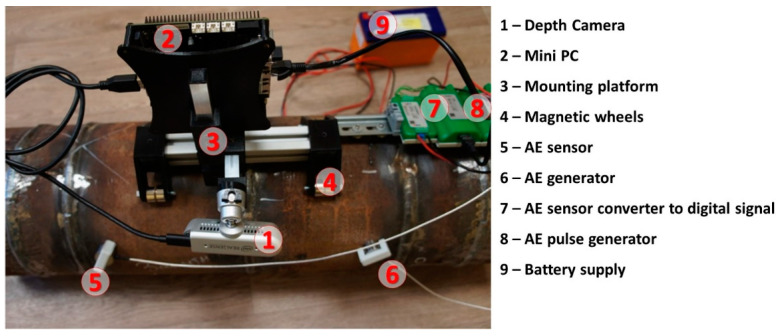
Experimental setup installed on the pipe.

The main point of the proposed study was to create intelligent technology to determine defects in steel pipeline welds. To solve this problem, the hardware platform and software tools were implemented. Our setup is based on an Odyssey X86J4105 microcomputer with 8 GB RAM, an SSD M.2 Kingston NV1 250 GB disk drive, an Intel RealSense D435i RGB-Depth camera, and two AE sensors ZET-601 (one generator and one sensor) driven by the ZET7104 platform. Using this setup, a dataset containing the two classes of images (nondefective and defective weld) was collected. Class 1 (nondefective weld) comprises 281 images in total with corresponded depth images (281 images, 250/20/11 train/test/validation), and class 2 (defective weld) comprises 71 corresponded depth images (71 images, 40/20/11 train/test/validation). Sample images are located in a ‘data’ folder at https://github.com/cybervllc/weldfs (accessed on 30 September 2022). The samples of AE data are located in a “data” folder at https://github.com/cybervllc/acoustic_emission (accessed on 30 September 2022). The experimental setup was built according to the 3D model prototype (see Figure 9).

**Figure 10:**
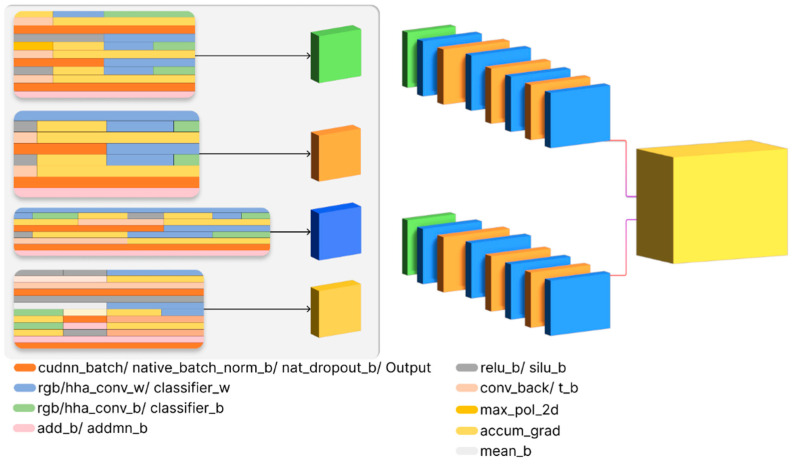
The core detail and layers of the DCNN for data fusion based on ResNet-18.

During the study, we trained several ResNet-18 models in the binary classification of defectiveness of welded steel pipeline joints. A workstation with an Nvidia RTX 2600 discrete graphics gas pedal with 12 GB of GDDR5 video memory, an Intel Core i5 11300 processor, and 16 GB of DDR4 RAM were used as the equipment. All models were implemented and trained with the Python functional programming language using the PyTorch library. The core detail of the proposed fusion architecture is shown in Figure 10.

**Figure 11:**
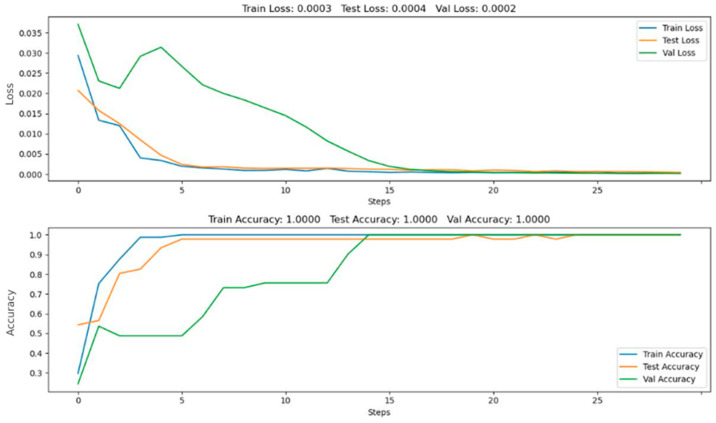
An example of the training results.

We considered the training processes of the residual network and its final characteristics for the case of classification. This consideration is based only on the image data of weld surface weld depths and scaleograms characterizing the AE method of NDT. All of the above cases were implemented using the ReLU and SiLU activation functions. Training was performed on the same number of measurements within the datasets. We trained the DCNN model with training hyperparameters, and for all cases they were as follows: epochs—25, batch size—32, learning rate—0.003, and momentum—0.9. An example of the training results is presented in Figure 11.

We conducted experiments with different ResNet-18-based models. The results and performance metrics are presented in Table 1.

As can be seen from Table 1, the proposed method demonstrates the highest accuracy out of all of the models but is the most computationally expensive and requires the greatest amount of time (including the formation of the logical output on the test data). However, these time intervals are within an acceptable range for the indicated problem. In addition, it should also be noted that, in two cases out of three, the structures of models using SiLU units showed higher accuracy but with a greater amount of time consumption. The exception is for conventional images of the control object, where ReLU proved to be a more efficient solution. It is also necessary to consider the general quality of the models for binary classification trained on different types of data characterizing a single object. The highest accuracy (86.74%) was reached by the model that learned on the acoustic emission spectra wavelet transform scalograms, and the lowest accuracy (66.2%) was reached by the model that learned on the data set of normal weld images. It is also worth noting the difference between the results of the method proposed in this study; it is 13.26% more accurate, at an additional time cost of 35 s. This fact shows that a large amount of data is not the only solution for stable and reliable identification of complex objects and processes, with the help of deep neural networks. The use of a small amount of data able to characterize the object of study using data fusion technology qualitatively and informationally and that allow us to achieve acceptable results yields areas where the collection of large amounts of data is difficult for various reasons. This fact opens new opportunities for deep learning to solve various applied problems. 

As part of the study, we also analyzed early data fusion using the example of a convolutional neural network. The following hyperparameters were used in the training of a neural network: Activation functions were ReLU and Softmax, dropout—none, batch size—32, learning rule—0.001, learning rate—0.0001.

**Figure 12:**
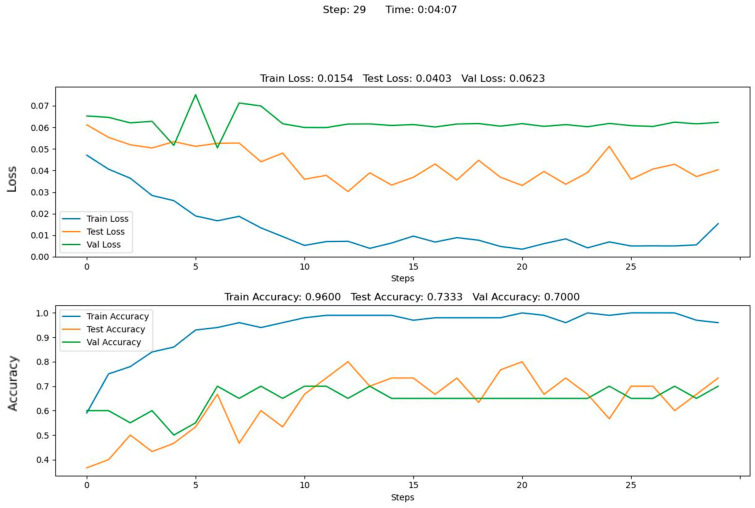
An example of training results for early fusion.

The learning quality metrics obtained, the model validation at the early fusion of RGB image data, the depth images of the measured object, and the AE data are presented in Figure 12.

This result is assumed to be primarily due to the divergence of the feature representation spaces extracted from RGB and depth images, as well as the AE Scaleogram. Therefore, the proposed technology used a complex of different-level data fusion methods considering different scenarios of preprocessing data of diverse modalities.

**Figure 13:**
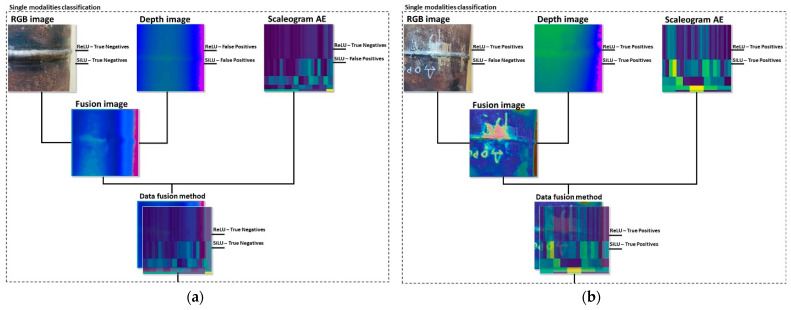
A result of classification for different approaches: (**a**) a test sample without a defect; (**b**) a test sample with a defect.

In addition, we tested the single modality approaches on samples with and without defects. Figure 13 presents examples of a defect detection procedure using different approaches.

## 4. Discussion

This study is based on the principle that proves the effectiveness of DCNNs on small datasets for solving applied problems. We considered the formation of data sets based not on a pure number of observations but from different data structures collected from heterogeneous sensors with a small number of measurements that are capable of specifically characterizing the object under study. As an example, this study considers the identification of defective weld joints in steel pipelines. Often, all possible defects of the weld made via electric arc welding are small rigid objects that can be enclosed both on the surface of the butt joint and inside it. Therefore, most of the time, technologies capable of characterizing the internal structure of the object studied are used as the main NDT approaches for the welded joints of steel pipelines.

However, with more careful thought about this problem, it can be said that there are some morphological characteristics for different defective areas of the weld, determining the permissibility of assigning a certain category to the pipeline when using it. This is because a more correct approach is not just the detection of some defective area by a nondestructive testing method, but a reliable identification of the detected violation, which allows a correct conclusion about the suitability of the work being performed to be made. A more detailed consideration of this issue within the automation framework of the surface inspection of welded joints using ML methods and computer vision algorithms shows that this problem will require the development of additional calculation functions and procedures that can expand our final representation of the control object in order to make the best decision on the presence or absence of a defect. This is a rather resource-consuming and complex approach, in addition to significantly increasing the computational complexity of the final processing algorithm. One possible solution could be to enrich the representation of the feature space. The feature space could be extracted by the model directly from the data by the model and thus be capable of forming a more complete spatial outline of these features for the reliable identification of the presence of defects. This approach will significantly reduce the resource costs necessary for preparation and significantly expand control object awareness in various measurement conditions. These factors will increase the efficiency of using deep learning models to support decision making in industrial processes.

## 5. Conclusions

We performed our study with the following considerations in mind. The idea of developing systems suitable for intelligent decision making concerning the surface quality of welded seams of steel gas pipelines is not new. Nevertheless, the problem of improving such systems using machine learning methods is very important. The current situation in the field of NDT shows a significant use of nondestructive testing methods, which are based on X-ray analysis. Such equipment has sufficient accuracy to detect defects but is inconvenient to use, including in difficult weather conditions. On the other hand, ultrasonic inspection equipment and various video cameras, which allow users to perform a video inspection of the surface of the pipeline welds, are quite widespread. However, it is quite difficult to establish an inspection system that combines the advantages of video surface inspection and the detection of hidden defects in welds. We theorize that one of the main problems for the successful dissemination of such systems is the insufficient development of integrated methods for fusing video and acoustic data. 

In the proposed study, we obtained some relevant results. A software and hardware system prototype for intelligent defects control in a steel pipeline welded joint was developed. A complex method for data fusion of various modalities was proposed to enrich the levels of features that characterize the measured object when evaluating the presence of a defect. A comparative analysis of the proposed method with approaches based on the single modalities data mining was carried out under the relatively small initial observations number condition. This analysis showed the effectiveness of the proposed technology under the same test conditions, which indicates the possibility of using deep learning models not only in the large presence of the same type of measurement data but also in reducing the number of necessary observations based on the simultaneous use of data from different sensors. The experiments showed a high accuracy in evaluating the defective and defect-free surfaces of welded seams of gas pipelines.

Among the main directions of future work based on this study, we would like to highlight the search for and the development of alternative models for efficient feature extraction and the development of concepts for the fusion of multidimensional data of various modalities at all fusion levels, considering increasing awareness of the control object. In addition, in the future, we would like to understand the boundary conditions and the relationship between intelligent decision making and deep learning models when changing the number of observations and the number of data modalities used for training. Furthermore, we are planning to extend the experience we gained to the implementation of a multiclass classification of defects in steel pipeline welds using the data fusion strategy.

## Figures and Tables

**Table 1:** Quality metrics of different model approaches.

Model	Accuracy	Error	Sensitivity	Specificity	Precision	False Positive Rate	Time
ResNet-18/ReLU/image	67.58	0.324	87.4	45.8	62.1	0.542	1 min 12 s
ResNet-18/SiLU/image	66.2	0.338	86.8	44.8	60.8	0.552	1 min 19 s
ResNet-18/ReLU/depth	78.31	0.217	88.9	67.1	73.15	0.329	1 min 5 s
ResNet-18/SiLU/depth	79.45	0.205	89.1	69.18	74.2	0.308	1 min 7 s
ResNet-18/ReLU/AE	84.1	0.159	100	67.74	82.97	0.322	1 min 24 s
ResNet-18/SiLU/AE	86.74	0.132	100	72.24	84.13	0.277	1 min 32 s
Our Method	100	0	100	100	100	0	2 min 7 s

## Data Availability

Not applicable.
